# Time to HAART Initiation after Diagnosis and Treatment of Opportunistic Infections in Patients with AIDS in Latin America

**DOI:** 10.1371/journal.pone.0153921

**Published:** 2016-06-07

**Authors:** Brenda Crabtree-Ramírez, Yanink Caro-Vega, Bryan E. Shepherd, Beatriz Grinsztejn, Marcelo Wolff, Claudia P. Cortes, Denis Padgett, Gabriela Carriquiry, Valeria Fink, Karu Jayathilake, Anna K. Person, Catherine McGowan, Juan Sierra-Madero, Caribbean, Central and South America Network for HIV Epidemiology (CCASAnet), of the International Epidemiologic Databases to Evaluate AIDS (IeDEA) Program

**Affiliations:** 1 Instituto Nacional de Ciencias Médicas y Nutrición, Salvador Zubirán, Infectious Diseases Department. Mexico City, Mexico; 2 Vanderbilt University, Department of Biostatistics, Nashville, TN, United States of America; 3 Instituto Nacional de Infectologia Evandro Chagas-Fiocruz, Rio de Janeiro, Brazil; 4 Universidad de Chile- Fundación Arriarán, Santiago, Chile; 5 Instituto Hondureño de Seguro Social and Hospital Escuela Universitario, Tegucigalpa, Honduras; 6 Instituto de Medicina Tropical Alexander von Humboldt, Lima, Peru; 7 Fundación Huésped, Investigaciones Clínicas, Buenos Aires, Argentina; 8 Vanderbilt University, Department of Medicine, Nashville, TN, United States of America; David Geffen School of Medicine at UCLA, UNITED STATES

## Abstract

**Background:**

Since 2009, earlier initiation of highly active antiretroviral therapy (HAART) after an opportunistic infection (OI) has been recommended based on lower risks of death and AIDS-related progression found in clinical trials. Delay in HAART initiation after OIs may be an important barrier for successful outcomes in patients with advanced disease. Timing of HAART initiation after an OI in “real life” settings in Latin America has not been evaluated.

**Methods:**

Patients in the Caribbean, Central and South America network for HIV Epidemiology (CCASAnet) ≥18 years of age at enrolment, from 2001–2012 who had an OI before HAART initiation were included. Patients were divided in an early HAART (EH) group (those initiating within 4 weeks of an OI) and a delayed HAART (DH) group (those initiating more than 4 weeks after an OI). All patients with an AIDS-defining OI were included. In patients with more than one OI the first event reported was considered. Calendar trends in the proportion of patients in the EH group (before and after 2009) were estimated by site and for the whole cohort. Factors associated with EH were estimated using multivariable logistic regression models.

**Results:**

A total of 1457 patients had an OI before HAART initiation and were included in the analysis: 213 from Argentina, 686 from Brazil, 283 from Chile, 119 from Honduras and 156 from Mexico. Most prevalent OI were Tuberculosis (31%), followed by *Pneumocystis* pneumonia (24%), Invasive Candidiasis (16%) and Toxoplasmosis (9%). Median time from OI to HAART initiation decreased significantly from 5.7 (interquartile range [IQR] 2.8–12.1) weeks before 2009 to 4.3 (IQR 2.0–7.1) after 2009 (p<0.01). Factors associated with starting HAART within 4 weeks of OI diagnosis were lower CD4 count at enrolment (p-<0.001), having a non-tuberculosis OI (p<0.001), study site (p<0.001), and more recent years of OI diagnosis (p<0.001).

**Discussion:**

The time from diagnosis of an OI to HAART initiation has decreased in Latin America coinciding with the publication of evidence of its benefit. We found important heterogeneity between sites which may reflect differences in clinical practices, local guidelines, and access to HAART. The impact of the timing of HAART initiation after OI on patient survival in this “real life” context needs further evaluation.

## Introduction

Although universal access to antiretroviral therapy in Latin America has been accomplished in most countries of the region, a large proportion of HIV-infected individuals still present late for care and therefore initiate HAART with low CD4 counts and/or with an AIDS-defining event (ADE) [1]. This population is more likely to progress to advanced stages of clinical AIDS, have poor immunologic response, and die [2–5]. The optimal timing of initiation of HAART in patients presenting with an ADE was debated in the early HAART era because of concerns of increased adverse outcomes such as overlapping toxicities of both treatments and risk of immune reconstitution inflammatory syndrome (IRIS) [6]. In 2009, the AIDS Clinical Trials Group (ACTG protocol 5164) reported that starting antiretroviral therapy within the first 30 days after a diagnosis of non-tuberculosis opportunistic infections (OI) was associated with a lower rate of AIDS progression and death, compared to delaying initiation of HAART, without an increase in adverse outcomes [7]. Other observational studies performed after ACTG 5164 support the early initiation of HAART after an ADE [8]. Moreover, recent clinical trials demonstrated a survival benefit in patients with HIV and tuberculosis with CD4 counts <50 cells/mm^3^ who initiate HAART early [9–12]. Furthermore, in a mathematical model of HIV disease to project the short- and long-term survival and costs of treating HIV-infected patients who present to care with acute OIs in the United States, early HAART initiation was suggested to be cost-effective [13]. Therefore most international guidelines currently recommend HAART initiation as early as possible after an ADE [14]. However, there is a lack of information of how in “real life” settings these recommendations are followed. In order to evaluate the implementation of this practice in individuals presenting with OIs, we aimed to evaluate the temporal trends of the time from a first diagnosis of OI to the time of starting HAART in HIV-positive treatment-naïve adults from the Caribbean, Central, and South America Network for HIV Epidemiology (CCASAnet) cohort.

## Methods

### Cohort

The Caribbean, Central, and South America Network (http://ccasanet.vanderbilt.edu/) has been described elsewhere [15]. CCASAnet is a consortium of HIV clinics from 7 countries (Argentina, Brazil, Chile, Haiti, Honduras, Mexico and Peru) and is a member cohort of the International Epidemiologic Databases to Evaluate AIDS (IeDEA; www.iedea.org). Five CCASAnet sites who routinelly collect clinical endpoints, contributed to this study: Fundación Huesped, Buenos Aires, Argentina (FH-Argentina); Instituto Nacional de Infectologia Evandro Chagas-Fiocruz, Rio de Janeiro, Brazil (FC- Brazil); Fundación Arriarán, Santiago, Chile (FA-Chile); Instituto Hondureño de Seguridad Social and Hospital Escuela, Tegucigalpa, Honduras (IHSS/HE-Honduras) and Instituto Nacional de Ciencias Médicas y Nutrición Salvador Zubirán, Mexico City, Mexico (INCMNSZ-Mexico).

### Study population

HIV-infected individuals were included if they were at least 18 years of age at enrolment from January 2001 to January 2012, and had an AIDS-defining opportunistic infection according to either WHO or CDC case definitions [16–17] before HAART initiation and no more than 30 days before entrance to care. Patients were divided into two groups based on previous studies evaluating the timing of HAART introduction [9–11]: those who initiated HAART within 4 weeks after the OI diagnosis (early HAART, EH) and those who initiated later than 4 weeks (delayed HAART, DH). All AIDS-related opportunistic infections were included. In patients with more than one opportunistic infection, the date of the first reported event was used.

### Statistical analysis

The probability of starting HAART over time for OIs occurring before and after January 1, 2009 was estimated using Kaplan-Meier curves. Additional analyses considered OIs due to tuberculosis (TB) or cryptococcal meningitis (CM). Factors associated with early HAART initiation (starting within 4 weeks after the diagnosis of OI) were estimated by logistic regression. Trends in the median time since the OI to HAART initiation according to year of OI were estimated with median regression controlling for gender, age, route of HIV transmission, site, CD4 count at enrolment, calendar year of OI, and whether the OI was tuberculosis or not. Continuous variables such as age, CD4 count at enrolment and calendar year were included in the regression models using restricted cubic splines. Statistical analyses were performed using R version 2.11.1 and analysis scripts are available at http://biostat.mc.vanderbilt.edu/ArchivedAnalyses.

### Ethics statement

Institutional ethics review boards from all sites and the CCASAnet Data Coordinating Center at Vanderbilt University, Nashville, TN, U.S.A. approved the project, waiving the requirement for individual patient informed consent.

## Results

### Study population

**Fig 1:**
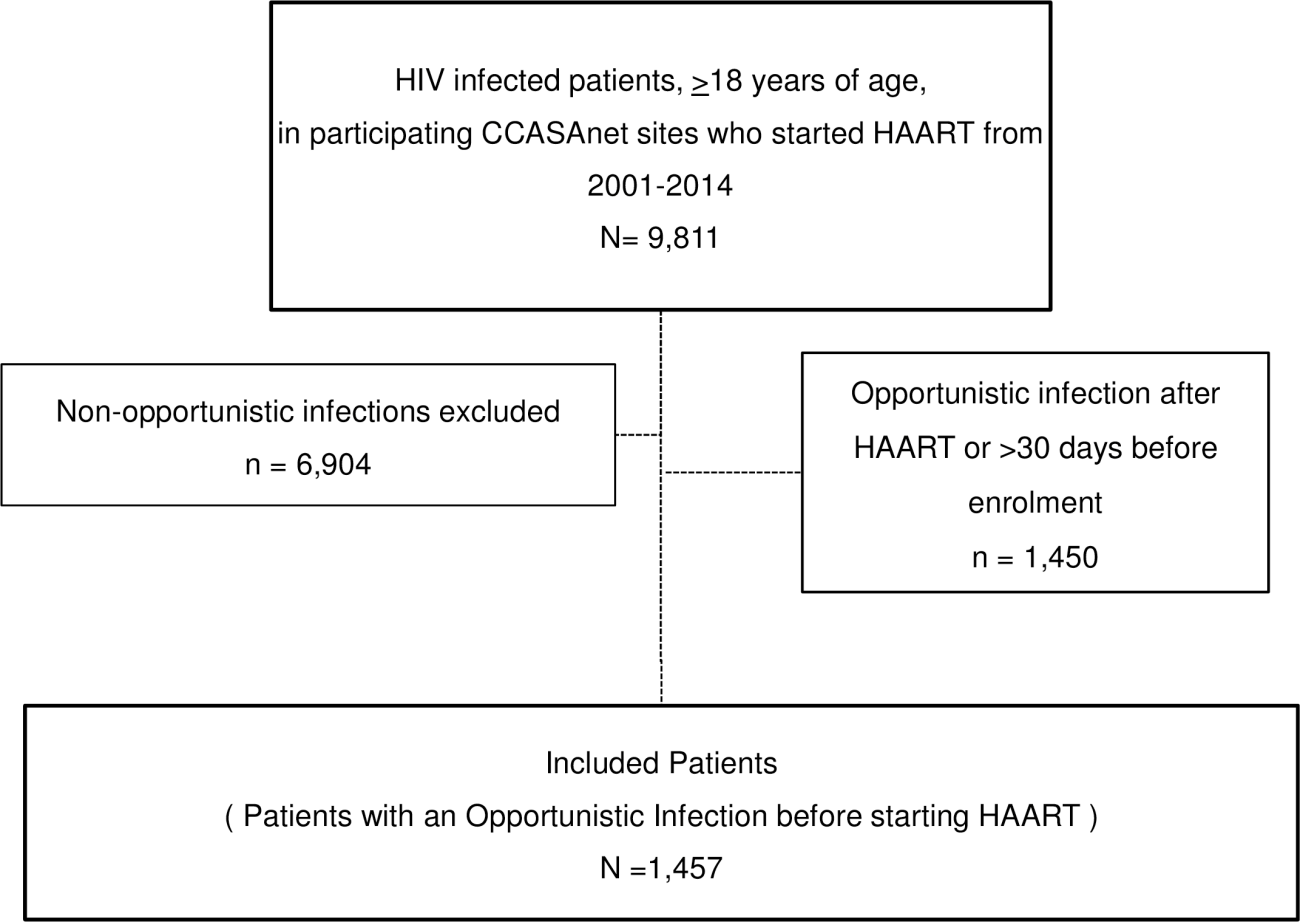
Elegibility of patients for analysis.

From a total of 12248 eligible patients, 1457 had an OI after enrolment but prior to HAART initiation, all of whom were included in analyses (Fig 1). FH-Argentina contributed 213 patients, FC-Brazil 686, FA-Chile 283, IHSS/HE-Honduras 119, and INCMNSZ-Mexico 156. Baseline characteristics of the study population are shown in Table 1. The median age at OI in the combined group was 36 years (interquartile range: 30–43); 77% were male and heterosexual transmission was the probable route of transmission for 45% of patients, although with varying proportions across sites. The median CD4 count at enrolment was 68 cells/mm^3^ with a high proportion missing CD4 count at enrolment, ranging from 26% to 62%. The different OIs and their frequencies by site are listed in Table 2. Tuberculosis was the most frequent OI, representing 32% of all OI cases (n = 453) followed by *P*. *jiroveci* pneumonia (n = 350), esophageal candidiasis (n = 237), and toxoplasmosis (n = 133). There were 51 cases of cryptococcal meningitis.

**Table 1:** Baseline patient characteristics according to site, and combined.

	FH-	FC-	FA-	IHSS/HE-	INCMNSZ -	Combined
	Argentina	Brazil	Chile	Honduras	México	(n = 1457)
	(n = 213)	(n = 686)	(n = 283)	(n = 119)	(n = 156)	
**Age (years)**	34 (30–40)	37 (30–44)	36 (31–44)	36 (30–42)	35 (28–42)	36 (30–43)
**Male**	165 (77.45%)	509 (74.2%)	257 (90.8%)	64 (53.7%)	138 (88.46%)	1133 (77.76%)
**CD4 at enrolment**						
**(cells/mm**^**3**^**)**	62 (21–122)	87 (35–252)	55 (20–173)	80 (46–147)	46 (17–106)	68 (25–197)
**Missing, n (%)**	132 (61.9%)	351(51.17%)	123 (43.46%)	39 (32.77%)	40 (25.64%)	643 (45.5%)
**TB cases**	27 (12.6%)	340 (49.56%)	21 (7.42%)	26 (21.8%)	40 (25.6%)	454 (31.16%)
**Cryptoccocal meningitis cases**	16 /7.51%)	14 (2.04%)	11 (3.8%)	8 (6.7%)	2 (1.26%)	51 (3.5%)
**Route of Transmission**						
Heterosexual	98 (46.01%)	345 (50.29%)	92 (32.51%)	74 (62.18%)	53 (33.97%)	662 (45.44%)
MSM	73 (34.27%)	216 (31.49%)	188 (66.43%)	4 (3.36%)	94 (60.26%)	575 (39.46%)
Other	35 (16.43%)	15 (2.19%)	2 (0.71%)	0 (0%)	5 (3.21%)	57 (3.91%)
Unknown	7 (3.29%)	110 (16.03%)	1 (<1%)	41 (34.45%)	4 (2.56%)	163 (11.19%)
**HAART regimen, n (%)**						
NNRTI	113 (53.05%)	524 (76.38%)	249 (87.99%)	116 (97.48%)	120 (76.9%)	1122 (77.01%)
PI	90 (42.25%)	120 (17.49%)	25 (8.83%)	1 (0.84%)	31 (19.87%)	267 (18.33%)
Other	10 (4.69%)	42 (6.12%)	9 (3.18%)	2 (1.68%)	5 (3.21%)	68 (4.67%)
**Time since OI and HAART in weeks**	7.4 (3-7- 16.6)	5.4 (3–10)	4.4 (1.1–10.9)	2.6 (0.7–4.9)	5.3 (2.7–8.3)	5.1 (2.3–10.1)
**Patients in early HAART group, n (%)**	59 (27.7%)	241 (3513%)	136 (48.06%)	81 (68.07%)	63 (40.38%)	580 (39.%)

MSM = Men who have sex with men; NNRTI = non-nucleoside reverse transcriptase inhibitor; PI = protease inhibitor; OI = opportunistic infection; Early HAART group = those who initiated HAART within 4 weeks after diagnosis of OI.

**Table 2:** Number of cases of each opportunistic infection reported according to site and combined.

Disease	FH-Argentina n = 213 (%)	FC-Brazil n = 686 (%)	FA-Chile n = 283 (%)	IHSS/HE-Honduras n = 119 (%)	INCMNSZ- Mexico n = 156 (%)	Combined n = 1457 (%)
Tuberculosis	27 (12.7)	340 (49.6)	21 (7.4)	26 (21.8)	40 (25.6)	454 (31.2)
Pneumocystis	53 (24.9)	124 (18.1)	125 (44.2)	10 (8.4)	35 (22.4)	347 (23.8)
Invasive candidiasis	24 (11.3)	84 (12.2)	91 (32.2)	10 (8.4)	27 (17.3)	236 (16.2)
Toxoplasmosis	20 (9.4)	49 (7.1)	9 (3.2)	42 (35.3)	6 (3.8)	126 (8.6)
Recurrent pneumonia	45 (21.1)	3 (0.4)	1 (0.4)	17 (14.3)	0 (0.0)	66 (4.5)
Cryptococcal meningitis	16 (7.5)	14 (2.0)	11 (3.9)	8 (6.7)	2 (1.3)	51 (3.5)
Severe recurrent herpes	0 (0.0)	21 (3.1)	8 (2.8)	0 (0.0)	2 (1.3)	31 (2.1)
Cytomegalovirus (CMV)	2 (0.9)	11 (1.6)	4 (1.4)	0 (0.0)	10 (6.4)	27 (1.9)
Histoplasmosis	7 (3.3)	6 (0.9)	0 (0.0)	6 (5.0)	8 (5.1)	27 (1.9)
CMV retinitis	6 (2.8)	10 (1.5)	2 (0.7)	0 (0.0)	7 (4.5)	25 (1.7)
Cryptosporidiosis	3 (1.4)	5 (0.7)	3 (1.1)	0 (0.0)	4 (2.6)	15 (1.0)
Atypical mycobacterium	3 (1.4)	7 (1.0)	2 (0.7)	0 (0.0)	2 (1.3)	14 (1.0)
Isosporiasis	2 (0.9)	7 (1.0)	3 (1.1)	0 (0.0)	1 (0.6)	13 (0.9)
Encephalopathy	4 (1.9)	0 (0.0)	0 (0.0)	0 (0.0)	8 (5.1)	12 (0.8)
Progressive multifocal leukoencephalopathy	1 (0.5)	5 (0.7)	2 (0.7)	0 (0.0)	2 (1.3)	10 (0.7)
Coccidioidomycosis	0 (0.0)	0 (0.0)	1 (0.4)	0 (0.0)	0 (0.0)	1 (0.1)
Salmonellosis	0 (0.0)	0 (0.0)	0 (0.0)	0 (0.0)	1 (0.6)	1 (0.1)

### Timing of HAART initiation after an OI and temporal trends

**Fig 2:**
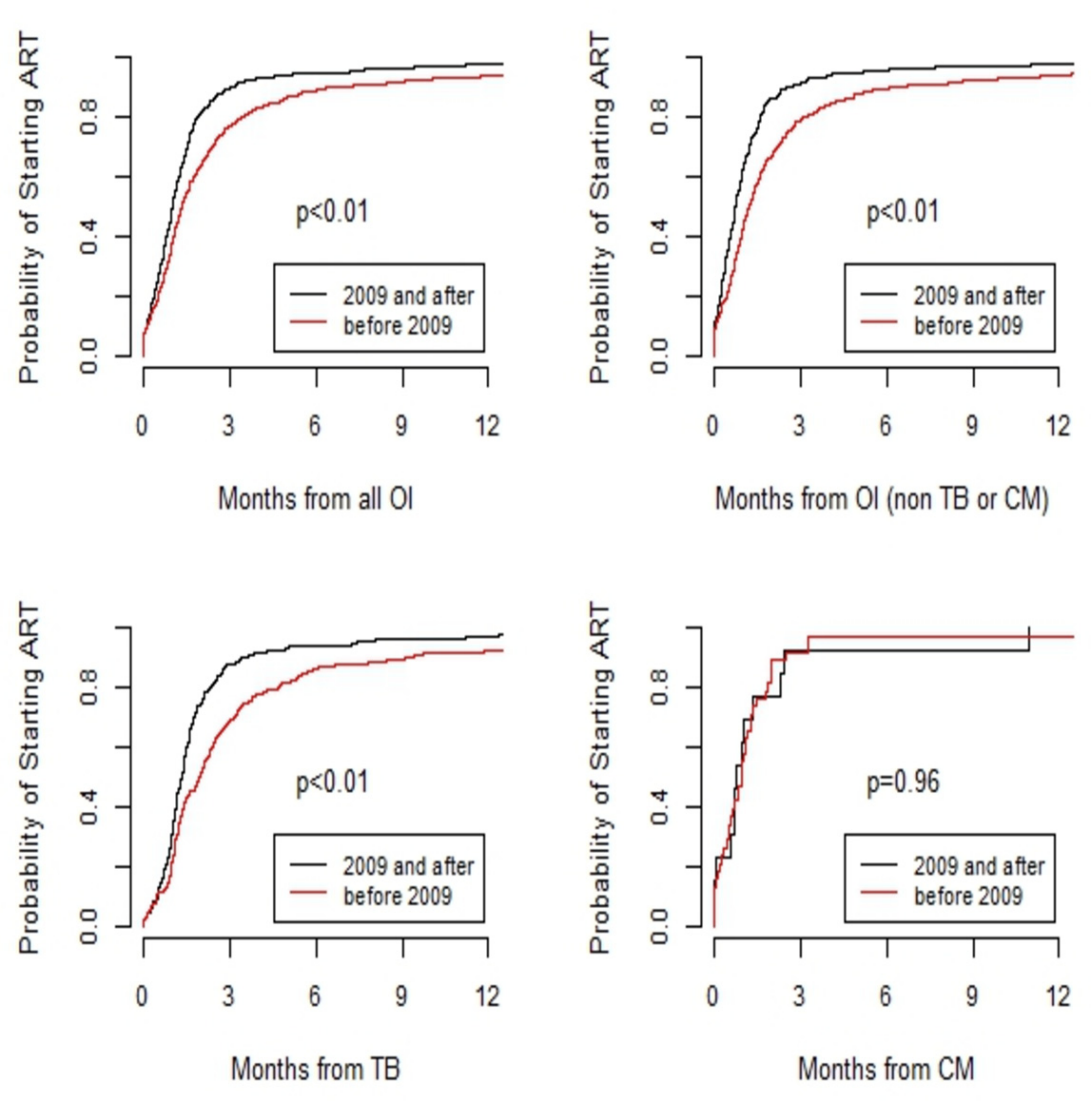
**Probability of starting HAART after all opportunistic infections** (A); opportunistic infections excluding tuberculosis and cryptoccocal meningitis (B); tuberculosis (C) or cryptococcal meningitis (D), before and after 2009.

The median time to HAART initiation after an OI was 5.1 weeks, and varied significantly between sites (from 2.6 weeks in IHSS/HE-Honduras to 7.4 weeks in FH-Argentina). Overall, 40% of patients started HAART within 4 weeks of their OI diagnosis; the percentage of individuals in the early HAART group also varied substantially between sites (from 28% in FH-Argentina to 68% in IHSS/HE-Honduras). The median time since the occurrence of an OI to HAART initiation was 5.7 weeks before 2009, and decreased to 4.3 weeks after that year (p<0.01). The probability of starting HAART over time after diagnosis with any OI, and after a diagnosis with TB or cryptococcal meningitis, is shown in Fig 2. In all cases, except for cryptococcal meningitis, the time to starting HAART was statistically shorter after 2009.

**Fig 3:**
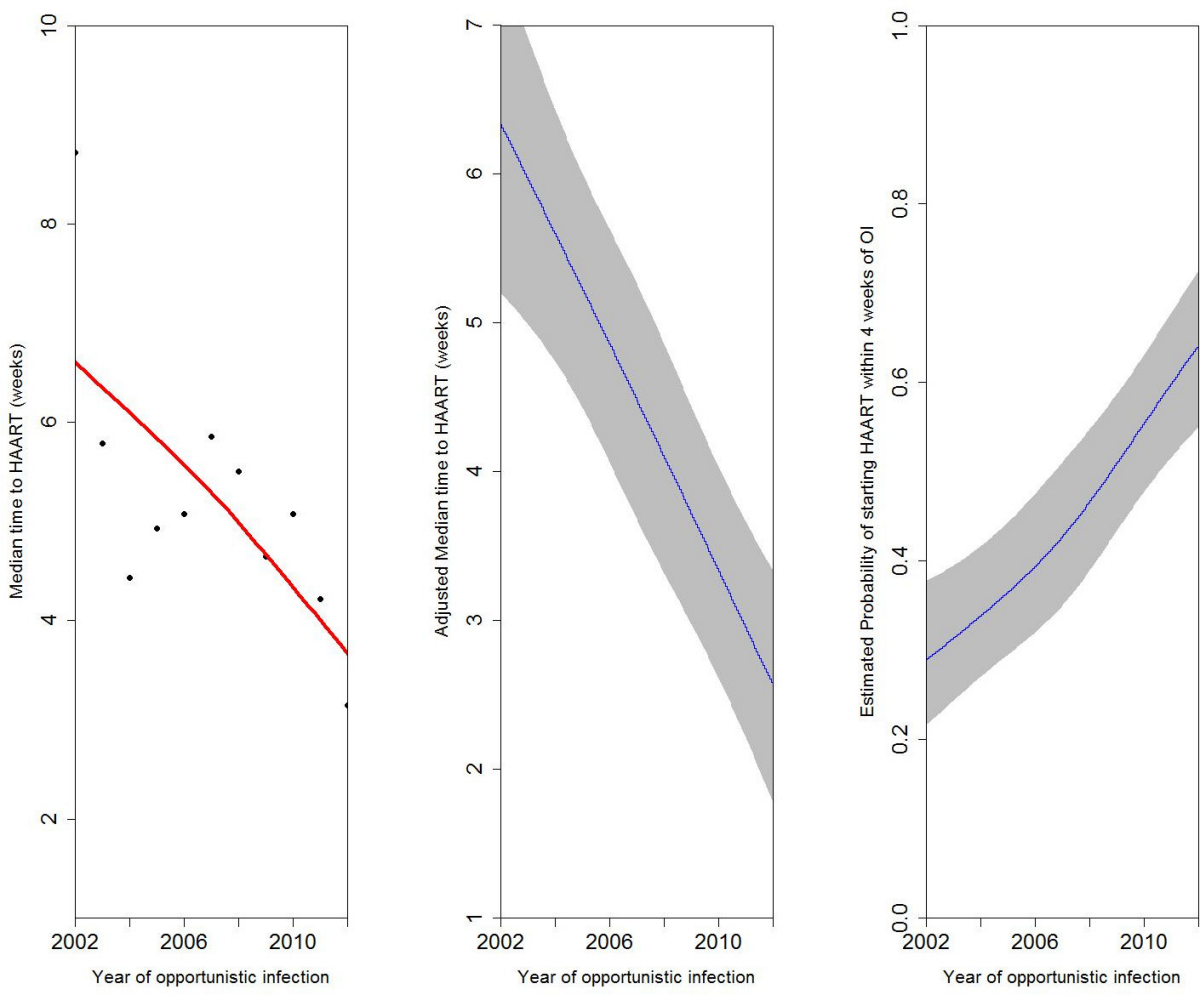
Trends over time in HAART initiation after an opportunistic infection (OI). A) Median time to HAART initiation over time (black dots are the median times according to calendar year, red line is the adjusted model. B) Trend across time adjusting for age, gender, route of HIV transmission, and CD4 count at enrolment, and tuberculosis diagnosis. C) Probability of starting HAART within 4 weeks of OI diagnosis over time adjusting for age, gender, route of HIV transmission, and CD4 count at enrolment, and tuberculosis diagnosis.

Fig 3 shows trends over calendar year in the time to HAART initiation after an OI. Panel A shows the median time to HAART initiation per year. Panel B shows the median time to HAART initiation per year after controlling for study site, age, gender, route of HIV transmission, and CD4 count at enrolment to care, and TB diagnosis. Panel C shows the estimated probability of starting HAART within 4 weeks of diagnosis with an OI as a function of calendar year, after controlling for the same variables. The results of all three panels are consistent and demonstrate a strong temporal trend towards earlier initiation of HAART after diagnosis of an OI (p≤0.01 for each analysis).

### Factors associated with early HAART initiation

Factors associated with starting HAART within the first 4 weeks after OI diagnosis are shown in Table 3. In addition to more recent calendar year, a lower CD4 count at enrolment was associated with being more likely to start HAART early. Patients from the site in Honduras as compared to Brazil also had an increased probability of being early HAART initiators (p-value<0.001). After controlling for other factors, patients diagnosed with TB were less likely to start HAART within 4 weeks than patients diagnosed with other OIs. Factors associated with early HAART initiation were similar when the time from OI to HAART initiation was kept as a continuous variable and analyzed with median regression (data not shown).

**Table 3:** Adjusted logistic model for factors associated with starting HAART within the first 4 weeks after opportunistic infection.

	Odds Ratio	(95% Confidence Interval)		p- value
**Age (years)**				
20	1			
30	1.02	0.88	1.19	0.78
40	1.05	0.77	1.42	
50	1.07	0.67	1.7	
**Male gender**	1.04	0.44	2.47	0.92
**Route of transmission**				
Heterosexual	1			0.427
MSM	0.84	0.57	1.23	
Other	1.09	0.42	2.86	
Unknown	1.49	0.83	2.64	
**CD4 count (cells/mm**^**3**^**)**				
25	1			**<0.001**
50	0.94	0.92	0.96	
100	0.83	0.77	0.89	
150	0.73	0.65	0.83	
200	0.64	0.54	0.77	
**TB**	0.34	0.24	0.51	
**CM**	0.94	0.39	2.23	0.88
**Year of OI**				
2002	1			**<0.001**
2004	1.06	0.83	1.36	
2006	1.16	0.72	1.85	
2008	1.42	0.83	2.44	
2010	1.98	1.19	3.3	
2012	2.83	1.63	4.9	
**Site**				
FC-Brazil	1			**<0.001**
FH-Argentina	0.69	0.38	1.26	
FA-Chile	1.58	0.99	2.54	
IHSS/HE-Honduras	4.48	2.32	8.65	
INCMNSZ- México	0.72	0.46	1.14	

MSM = Men who have sex with men; TB = tuberculosis; CM = cryptoccocal meningitis; OI = opportunistic infection.

## Discussion

The main purpose of this work was to describe the changes that have occurred over time in Latin America regarding the timing of HAART initiation after diagnosis of an opportunistic infection. We found that the time between OI diagnosis and starting HAART has decreased significantly in recent years, but that there is substantial heterogeneity between sites. After the year 2009 most international guidelines recommended HAART initiation as early as possible after an OI diagnosis [14]. In concordance with this recommendation we found a statistically significant higher probability of early HAART initiation after 2009 than before. The practice did not change immediately in 2009, but rather was a gradual change, and appears to have been occurring prior to 2009, although it has perhaps accelerated in more recent years.

Because of potentially different clinical implications compared to other OIs, tuberculosis cases were analyzed separately but also included in the overall analysis. Patients diagnosed with tuberculosis, as opposed to other OIs, were more likely to delay HAART initiation. The evidence regarding the optimal time for starting HAART after a tuberculosis event has been clearly documented in several important clinical trials [9–11]. In each of these, the lower the CD4 count at TB diagnosis, the higher the benefit of starting HAART earlier. Even though TB-IRIS occurs more frequently with lower CD4 count when starting HAART, mortality is higher if treatment is deferred [9–11].

In a recent observational study conducted in Uganda in patients with HIV and tuberculosis co-infection and CD4 counts less than 50 cells/mm^3^, the proportion starting HAART within 14 and 30 days of diagnosis of tuberculosis increased from 7% to 14% and from 14% to 86%, respectively, over the period of observation [18]. The ICONA cohort in high-income countries from Europe recently found a significant increase in early HAART initiation after a documented OI in three different periods of time between 1996 and 2013 [19]. Factors associated with early initiation of HAART in this context were recent calendar year, lower CD4 count, and a recent HIV diagnosis, which is consistent with our results.

In contrast to the evidence existing for outcomes according to time to HAART initiation after occurrence of tuberculosis, the evidence through clinical trials for other OIs is scarce. The multisite ATCG 5164 study published by Zolopa and colleagues in 2009 demonstrated a statistically favorable composite outcome (proportion of individuals alive, free of AIDS and with undetectable viral load) at 48 weeks after enrolment in patients presenting with an OI (including cryptococcal meningitis, but excluding TB) in whom HAART was initiated in the first 2 weeks in comparison to those who initiated between 6 to 12 weeks, especially in more immunosuppressed patients with CD4 counts less than 50 cells/mm^3^ [7]. A more recent prospective study performed only in Spain (PISCIS cohort) evaluated the impact of timing of HAART on disease progression and death among HIV-infected patients with an AIDS-defining event [8]. The results showed that deferring HAART was significantly associated with faster progression to a new AIDS-defining event or death overall [8].

The recently published START study results support the initiation of HAART regardless of CD4 count [20], and demonstrated the significant benefits for initiating at CD4 counts >500 cells/mm^3^ versus deferring until CD4 counts fall below 350 cells/mm^3^ (which was the previous recommendation according to WHO guidelines published in 2010) [12,14]. In the context of an OI, international guidelines have recommended initiation of HAART as early as possible since 2009, except in certain circumstances such as cryptococcal meningitis [6,21], yet evaluation of the translation of this recommendation to routine care of patients is limited. In our study, trends over time for reducing the time from OI diagnosis to HAART initiation were clearly significant when all OIs were included. However, we found no distinct trend in earlier HAART initiation after diagnosis of cryptococcal meningitis, perhaps in part due to evolving knowledge about the optimal timing of HAART in this circumstance; the number of cryptococcal meningitis cases included in our study was low (n = 51) which may have limited our power to detect temporal trends.

The heterogeneity of the timeline to HAART initiation after an OI between sites included in our cohort may reflect different patterns of adopting “state of the art” practice in each country. The diverse nature of specific OIs across sites may have driven physicians to differentially defer HAART (for example, the Brazilian site contributed half of the TB events in the study) due perhaps to differing concerns for IRIS. In the context of TB/HIV co-infection, IRIS has been analyzed in several studies. The findings suggest that the benefits of starting HAART in patients with very advanced disease might supersede the risks of mortality attributed to IRIS itself [11,22]. In that sense, prevalence of IRIS may vary between our sites and contribute to the heterogeneity of the results. Moreover, limited availability of diagnostic tests and infrastructure at some sites may delay OI diagnosis and therefore timing of HAART initiation. Finally, barriers to the access of HAART in such situations may differ between HAART programs in each region. Therefore, evaluations to describe the adoption of these recommendations in a “real life” context are crucial to identify potential areas for improvement.

We recognize strengths and limitations for our study. To our knowledge this is the first study in Latin America to describe the temporal trends of the time from a first diagnosis of OI to the time of starting HAART in HIV-infected treatment-naïve adults. However, even though patients from several Latin American countries were included, the generalizability and external validity of our results in the region are limited. Our cohort does not collect data on IRIS, which could have further added to the analysis. Opportunistic infections were not uniformly collected across sites and there may have been some missed or incorrect diagnoses. Finally, analysis of the impact of the timing of HAART initiation on mortality was not performed and warrants further evaluation.

## Conclusion

In this “real life” evaluation conducted in several sites in Latin America, the time from diagnosis of an OI to HAART initiation has decreased, coinciding with the publication of evidence of the benefits of early HAART initiation. In multivariable analyses there was a clear association of shorter time to HAART initiation in recent years of presentation. Other factors associated with early HAART initiation after an OI diagnosis were non-tuberculosis diagnosis and low CD4 counts at baseline. We found important heterogeneity between sites, which may reflect differences in clinical practices and resources, local guidelines, and access to HAART. The impact of the timing of HAART initiation after OI on patient survival in this “real life” context needs further evaluation.
